# Supplementary material for: Reconciling Mining with the Conservation of Cave Biodiversity: A Quantitative Baseline to Help Establish Conservation Priorities
Source: PLoS One. 2016 Dec 20;11(12):e0168348. doi: 10.1371/journal.pone.0168348 (PMC5173368; doi:10.1371/journal.pone.0168348)
Supplement: S1 Dataset — (ZIP) [file pone.0168348.s002.zip › Taxa/Serra Norte/SN_2013/N5S/Lista N5S-83.pdf]

CAVIDADE N5S-0083

Classe	Ordem	Fam/Outros	Gên/Outros	Espécie	S-U	U-S	Zon.
Annelida	Oligochaeta			jovem	1		E
Arachnida	Acari	Asternoseiidae		sp.84	X		E
Arachnida	Acari	Ixodidae		jovem	X		E
Arachnida	Acari	Veigaiidae		sp.1		X	E
Arachnida	Amblypygi	Phrynidae	*Heterophrynus*	*longicornis*	3	2	E/P
Arachnida	Araneae	Pholcidae	*Mesabolivar*	*aurantiacus*	X		E
Arachnida	Araneae	Pisauridae	*Thaumasia*	*velox*		2	E
Arachnida	Araneae	Scytodidae	*Scytodes*	jovem		2	E
Arachnida	Araneae	Theridiidae		jovem		X	E
Arachnida	Araneae	Theridiosomatidae	*Plato*	sp.1	X	X	E
Arachnida	Araneae	Trechaleidae		jovem		6	P
Arachnida	Pseudoscorpiones	Chthoniidae	*Pseudochthonius*	sp.1		X	E
Entognatha	Collembola	Cyphoderidae		sp.1	X		E
Entognatha	Collembola	Paronellidae		sp.1	X	X	E
Insecta	Blattodea			jovem		1	E
Insecta	Diptera	Tipulidae	Tipulinae	sp.	X	X	E
Insecta	Heteroptera	Hebridae	*Hebrus*	jovem	X		E
Insecta	Heteroptera	Veliidae	*Microvelia*	jovem	X		E
Insecta	Heteroptera	Veliidae	*Paravelia*	jovem		X	E
Insecta	Hymenoptera	Formicidae	*Crematogaster*	sp.1	X		E
Insecta	Isoptera	Termitidae	*Armitermes*	sp.		X	E
Insecta	Isoptera	Termitidae	*Nasutitermes*	sp.	X	X	E
Insecta	Orthoptera	Phalangopsidae	*Paraclodes*	sp.	26	1	E/P
Insecta	Orthoptera	Phalangopsidae	*Phalangopsis*	sp.	1	13	E/P
Insecta	Psocoptera			jovem		X	E
Insecta	Thysanura	Nicoletiidae		jovem	X		E
Amphibia	Anura			sp.	1	2	E
Mammalia	Chiroptera	Furipteridae	*Furipterus*	*horrens*	1		E
